# Spatiotemporal analysis of event‐related fMRI to reveal cognitive states

**DOI:** 10.1002/hbm.24831

**Published:** 2019-11-14

**Authors:** Jon M. Fincham, Hee Seung Lee, John R. Anderson

**Affiliations:** ^1^ Department of Psychology Carnegie Mellon University Pittsburgh Pennsylvania; ^2^ Department of Education Yonsei University Seoul Republic of Korea

**Keywords:** cognitive states, fMRI experiment, HSMM‐MVPA method

## Abstract

Cognitive science has a rich history of developing theories of processing that characterize the mental steps involved in performance of many tasks. Recent work in neuroimaging and machine learning has greatly improved our ability to link cognitive processes with what is happening in the brain. This article analyzes a hidden semi‐Markov model‐multivoxel pattern‐analysis (HSMM‐MVPA) methodology that we have developed for inferring the sequence of brain states one traverses in the performance of a cognitive task. The method is applied to a functional magnetic resonance imaging (fMRI) experiment where task boundaries are known that should separate states. The method is able to accurately identify those boundaries. Then, applying the method to synthetic data, we explore more fully those factors that influence performance of the method: signal‐to‐noise ratio, numbers of states, state sojourn times, and numbers of underlying experimental conditions. The results indicate the types of experimental tasks where applications of the HSMM‐MVPA method are likely to yield accurate and insightful results.

## 1 BACKGROUND

In cognitive science, our ability to construct theories of cognitive processes has outstretched our ability to adequately test the theories. Cognitive science theories now postulate sequences of processes, potentially progressing in parallel, that take fractions of a second. They postulate distributions on the durations of these processes. They allow for subjects to take alternative solution paths that can lead to the same result. Essentially, each time a subject undertakes to solve a problem they follow a different journey. In contrast to this theoretical richness, the classic measures of cognitive science are very coarse: whether an answer is correct, total time to perform a task, activation in a brain region, and so forth. These measures compress the journey taken on a problem into a single number. There are measures that do try to track the process, notably verbal protocols and eye movements. However, verbal protocols (e.g., Ericsson, 2006) provide descriptions at a temporal grain size much above the speed of cognition and have problems with their reliability. In principle, eye movements (e.g., Kowler, 2011) come closer to the speed of cognition, but this feature is typically not exploited and eye movement data tend to be aggregated into average measures.

Neuroimaging data on the other hand have the potential for illuminating and constraining detailed theories of cognitive processing. Their potential for analyzing moment‐by‐moment processing has certainly been recognized (Gonzalez‐Castillo et al., 2015; King & Dehaene, 2014). While the undertaking is not trivial, there have been advances toward bridging the gap between putative cognitive processes and the underlying neuromechanisms that mediate that processing (Palmeri, Love, & Turner, 2017). Techniques for linking behavioral and neural data and combining modeling data from multiple sources are being developed (Borst & Anderson, 2017; Cohen et al., 2017; Rubin et al., 2017; Turner, Forstmann, Love, Palmeri, & Van Maanen, 2017; Turner, Rodriguez, Norcia, McClure, & Steyvers, 2016).

With our own work in this area, we (e.g., Anderson, Betts, Ferris, & Fincham, 2010; Anderson, Lee, & Fincham, 2014; Anderson, Pyke, & Fincham, 2016; Anderson, Zhang, Borst, & Walsh, 2016) have developed a method that can track what is happening as a particular individual performs a specific task. The method combines spatial pattern matching with temporal pattern matching. The spatial pattern matching involves using multivoxel pattern‐analysis (MVPA—Norman, Polyn, Detre, & Haxby, 2006; Pereira, Mitchell, & Botvinick, 2009) to recognize distinct mental states from whole brain activation. The temporal pattern matching involves using hidden semi‐Markov models (HSMMs; semi‐Markov because the models allow the duration of a state to vary according to a density; Rabiner, 1989; Yu, 2010). Hutchinson, Niculescu, Keller, Rustandi, and Mitchell (2009) describe an application of similar Hidden Process models to parsing the temporal structure of fMRI data. Hidden Markov models (HMMs) have been used to identify distinct states in resting state data (e.g., Eavani, Satterthwaite, Gur, Gur, & Davatzikos, 2013; Suk, Wee, Lee, & Shen, 2016; Vidaurre, Smith, & Woolrich, 2017; Warnick et al., 2018). Shappell, Caffo, Pekar, and Lindquist (2019) compare HMMs with HSMMs and show that the HSMM approach, which is ours, is better capable of estimating state sojourn time.

**Figure 1:**
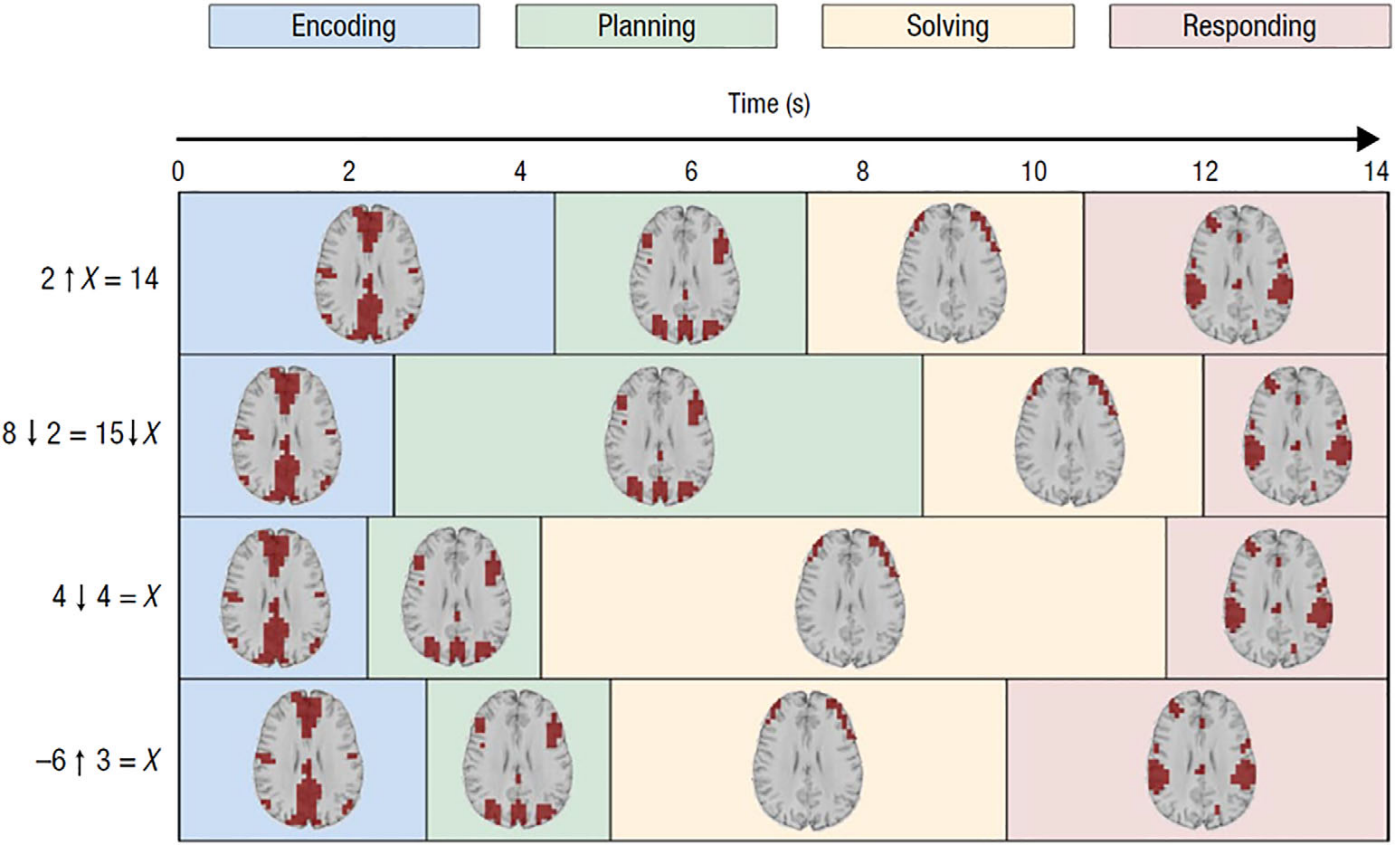
An illustration of the state sojourn times for four trials where different problems were solved. The four problems are given to the left where the arrows denote new mathematical operators that students had learned. Each problem has different cognitive demands, and all happened to take 14 s to solve. In each state, the axial slice (*z* = 28 mm at *x* = 0, *y* = 0 in Talairach space) highlights brain regions whose activation in that state is significantly greater than the average activation during the entire problem. Note brain images are displayed in radiological convention with left brain on the right *Source*: Anderson et al. (2015)

The HSMM‐MVPA method is able to take the brain activity over the course of individual experimental trials and parse it into a sequence of unique brain states and corresponding sojourn times. Each state, or brain signature, is simply a brain activation pattern that is roughly constant during the sojourn time. Figure 1 illustrates a recent application of this method to a novel mathematical task (Anderson, Pyke, & Fincham, 2016; Anderson, Zhang, et al., 2016). Each trial corresponded to solving a particular problem, beginning with the problem presentation and ending with the keying of a response. The method found evidence that when solving these problems, subjects traversed four sequential states, each corresponding to a unique brain signature (labeled here as Encoding, Planning, Solving, and Responding). In addition to estimating the brain signatures, the HSMM‐MVPA estimates the corresponding sojourn times for these states on each problem. The figure shows the parses of four problems that differed from one another in their cognitive demands, but that happened to all take 14 s (trial times ranged from 9 to 30 s). The different sojourn times for states across these problems reflect the different cognitive demands of each. Aggregating these trial‐by‐trial parses allowed us to understand how subjects were solving these problems in a way that we would not have been able to had we limited our analyses to total time spent solving the problems or aggregate brain activity over trials. For instance, Anderson, Pyke, & Fincham (2016) and Anderson, Zhang, et al. (2016) showed that duration of the Solving stage varied with the number of additions that participants had to perform.

That study and other related work have demonstrated the usefulness of the technique in analyzing imaging data. However, there are methodological issues that need to be addressed so that this method can be appropriately and successfully applied to imaging data. Consider again the data in Figure 1. How do we judge how well this method has actually captured the true state of affairs in these data? Would some number other than four states better represent the data? How do we define “better” and how do we evaluate such a claim? In that experiment (and similarly with other experiments in which we have applied the HSMM‐MVPA method), individual trials may take up to 30 s (on the order of 1 min in other tasks) to complete. Further, there typically is no clearly defined ground truth from which to begin evaluating the goodness of a particular *N*‐state model. The only verifiable trial markers are stimulus onset and the keying of a response: there are neither experimental task markers nor external cues that indicate when “planning” stops and “solving” begins for example. If there were such task markers, it would be a straightforward matter to measure the goodness of fit between predicted and actual state boundaries (and of course less need to apply the method in the first place, as we could directly analyze brain activity during such well‐defined periods).

The purpose of this article is to assess the veridicality of the state divisions that the HSMM‐MVPA method produces and to understand the factors that influence the accuracy of the method and usefulness of results. In the first part of this article, we apply the method to an fMRI experiment (Lee, Fincham, & Anderson, 2015) that, unlike the experiment shown in Figure 1, has well‐defined task markers that will provide a ground truth from which we can evaluate the goodness of model fits. In that context we describe the methodology, the notion of informed and uninformed models, and assessing model goodness using several metrics. This will serve to frame the issues important for using the technique to infer useful models.

In the remainder of the article, we extend the experimental results by applying the method to synthetic datasets that have been generated by differing ground truth state structures. We will consider a number of factors that influence how well the method performs, including signal‐to‐noise, numbers of states, state sojourn times, and numbers of conditions. The results will define a “sweet range” for various parameters of experimental tasks where applications of the HSMM‐MVPA method are likely to yield both accurate and insightful results.

## 2 HSMM‐MVPA APPLIED TO EXPERIMENTAL DATA: LEE ET AL. (2015)

### 2.1 Study description

**Figure 2:**
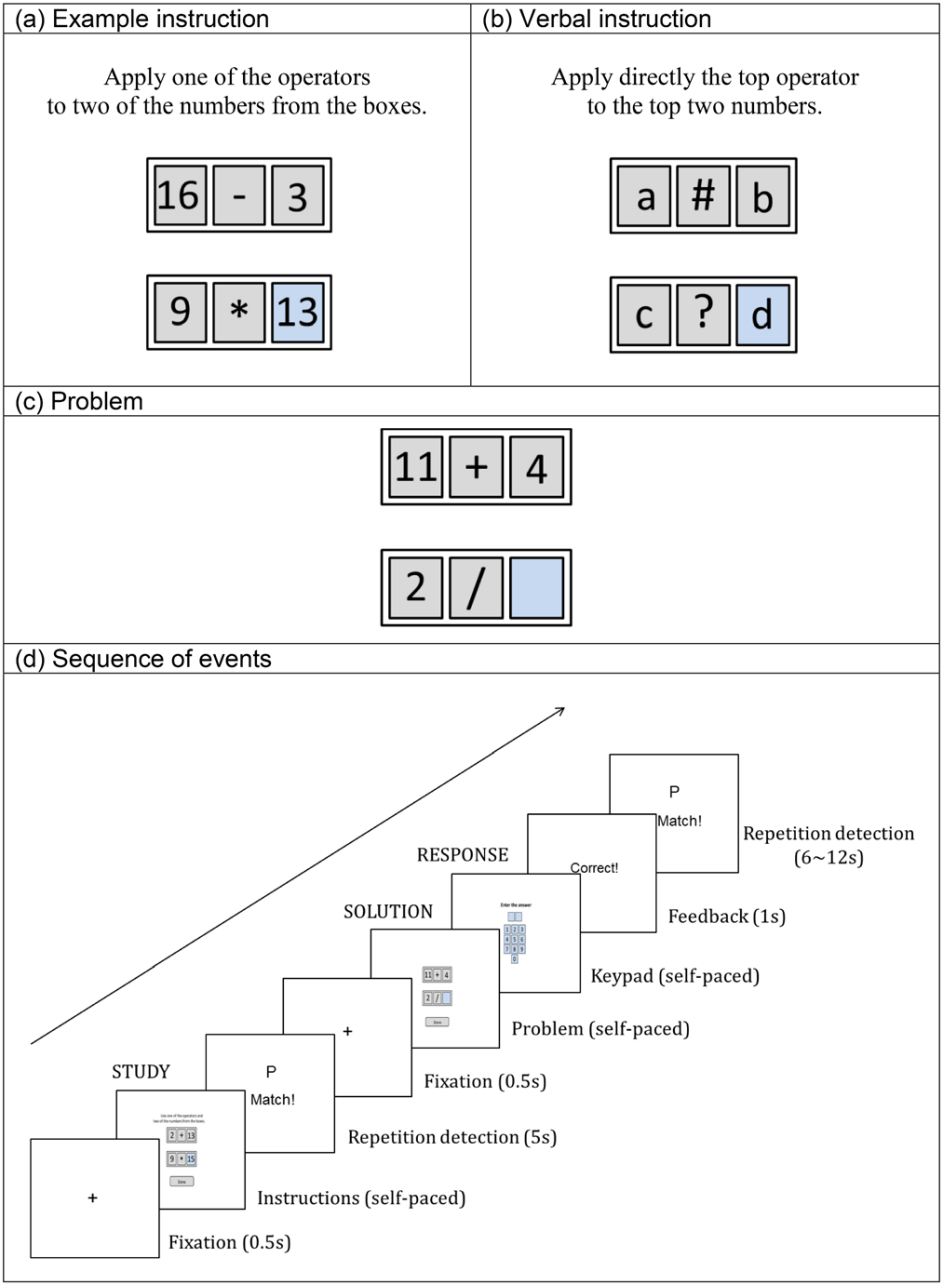
The Lee et al. (2015) experiment. (a) and (b) Examples of what subjects studied in the two conditions; (c) The common presentation of problems to solve; (d) The sequence of events in a trial

Figure 2 illustrates the two experimental conditions and experimental sequence used by Lee et al. (2015). In an fMRI scanner subjects alternated between studying a new arithmetic rule to fill in cell of a diagram and solving a problem where they had to apply the new rule (each rule was just applied once). While the textual and graphical complexity was constant, in some cases the critical information was in a graphical example (Figure 2a) of the rule and in others it was in a verbal instruction (Figure 2b) on how to apply the rule. After studying the instruction, participants had to apply it to a problem presented identically for either study condition (Figure 2c). This defined the two conditions of the experiment—Example and Verbal. Figure 2d illustrates the detailed structure of the experiment which we broke into five phases, for which we have recorded onset and offset:

1. Study: Self‐paced study ended by clicking a done button.
2. Delay: A fixed 5‐s duration repetition‐detection task and a half‐second fixation. In the repetition‐detection task, letters appeared on the screen at a rate of 1 every 1.25 s. Participants were told to click a match button whenever the same letter appeared twice consecutively. This was intended to discourage participants from extending their encoding of the instructions and to separate study and solution periods.
3. Solve: Self‐paced problem solution ended by clicking a done button.
4. Respond: A self‐paced response period that subjects had to enter their answer on a keypad followed by a 1 s feedback. Subjects were encouraged to have the answer ready to key quickly.
5. Detect: A 6–12 s repetition‐detection phase followed by the fixation that preceded the next problem. As with the repetition‐detection task in the delay period, letters appeared on the screen at a rate of 1 every 1.25 s. Participants were told to click a match button whenever the same letter appeared twice consecutively. This was intended to provide a baseline period of activity with minimal cognitive demand.

Adjacent behavioral phases within each experimental trial are distinct from one another in terms of cognitive demands. While a phase may be made up of just one or potentially more successive brain states, each transition from one behavioral phase to another should in theory be accompanied by a corresponding transition in brain state. The first question of interest is how well the HSMM‐MVPA method can do in discovering these behavioral phase boundaries.^1^

### 2.2 Behavioral data

**Figure 3:**
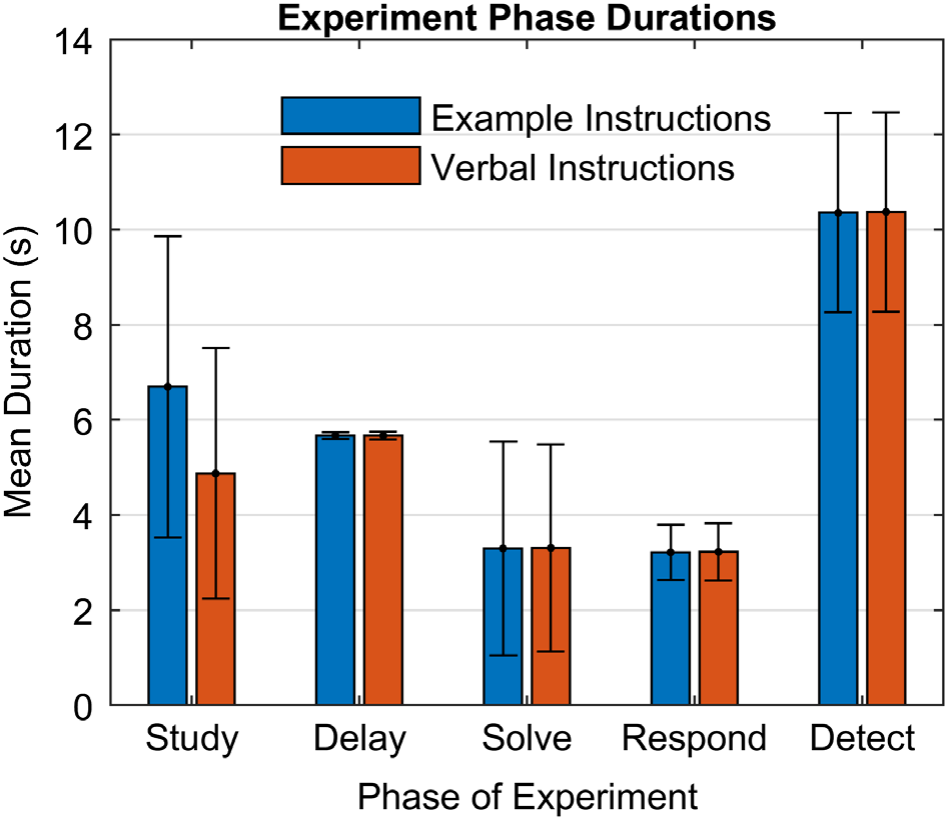
Mean durations with SD error bars of phases in the two conditions of Lee et al. (2015)

The experiment is described in detail in the original study. Here we will focus on describing the resulting data and its processing. Twenty subjects solved three blocks of 24 problems, equally divided into the two conditions. Looking at correct problems (96.8% in the Verbal condition and 93.5% in the Example) and excluding trials with problematic features,^2^ there were 654 Verbal problems and 632 Example problems. Individual trials varied from 18 to 56 s with a mean of 28.3 s and a *SD* of 4.8 s. Figure 3 shows the mean time (bars) subjects spent in the five phases of the two conditions and the trial‐to‐trial variability (SD error bars) in this time. The mean times for the two conditions are nearly identical except for the Study phase where subjects needed more time to process the example. There is low variability in the duration of the Delay phase or the Respond Phase. In the case of the Delay Phase the low variability reflects the fixed timing of the experimental procedure; in the case of Respond Phase this is because the demands of keying in a two‐digit number are fairly constant. The high variability in the case of the Study and Solve phases reflects variability in how long subjects took to understand the instructions and solve the problem. The high variability in the Detect phase reflects the experimental procedure (Figure 2d), which involved variability (jittering) in the duration of this phase.

### 2.3 Imaging data processing

#### 2.3.1 Overview

The data processing stream first passes whole brain fMRI activity through standard fMRI processing steps and then converts this into a form appropriate for application of HSMM‐MVPA: a small number of orthogonal dimensions of activity where the activity has been deconvolved so that it represents processing at a point in time, which generated the downstream BOLD response. Below we lay out the steps in that processing.

#### 2.3.2 Imaging processing

Images were acquired using gradient‐echo echo planar image (EPI) acquisition on a Siemens 3T Verio Scanner using a 32 channel RF head coil, with 2 s repetition time (TR), 30 ms echo time (TE), 79° flip angle, and 20 cm field of view (FOV). The experiment acquired 34 axial slices on each TR using a 3.2 mm thick, 64 × 64 matrix. This produces voxels that are 3.2 mm high and 3.125 × 3.125 mm^2^. The anterior commissure‐posterior commissure (AC‐PC) line was on the 11th slice from the bottom scan slice. Acquired images were preprocessed using AFNI (Cox, 1996; Cox & Hyde, 1997). Functional images were motion‐corrected using 6‐parameter 3D registration. All images were then slice‐time centered at 1 s and co‐registered to a common reference structural MRI by means of a 12‐parameter 3D registration and smoothed with a 6 mm full‐width‐half‐maximum 3D Gaussian filter to accommodate individual differences in anatomy. A functional mask was created using the common reference brain. For each scanning run (60 runs, 3 runs per subject × 20 subjects), in‐mask voxels were selected (47,315 in all) for processing. Within each run, BOLD response values were generated for each voxel time series by normalizing each to have mean value of 100. Lastly, signal drift was regressed out of each time series using a fourth‐degree polynomial.

#### 2.3.3 Deconvolution

The primary goal of deconvolution is to take the lagged and diffuse BOLD signal and extract an inferred activity signal for each time point, effectively temporally aligning signal and behavioral data. The resultant time series for each run from the image‐processing step was deconvolved by applying a Weiner filter (Glover, 1999) with a hemodynamic response function to produce the estimate of the underlying activity signal for each 2‐s time point of the run. The hemodynamic function is the SPM difference of gammas (Friston, Ashburner, Kiebel, Nichols, & Penny, 2011: gamma(6,1)‐gamma(16,1)/6). The Weiner filter also requires specification of a noise‐to‐signal parameter and we used a value of .1, as we have in other applications. The Appendix shows that a wide range of plausible noise‐to‐signal values yields similar results.

#### 2.3.4 Principle component analysis

**Figure 4:**
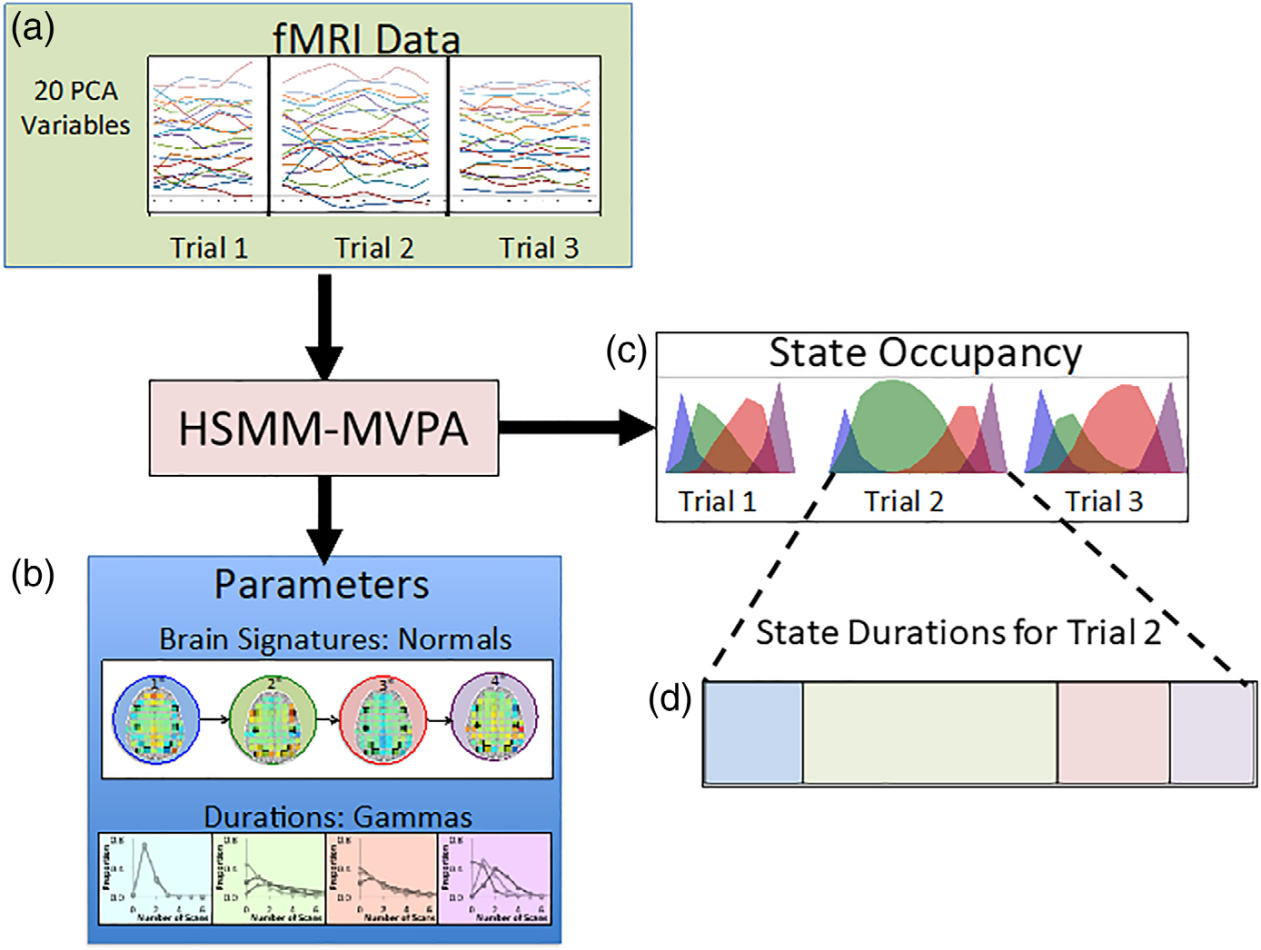
An illustration of the HSMM‐MVPA method for a 4‐state solution: (a) The input is the brain activity on each trial, whose dimensionality has been reduced by a principal component analysis; (b) two types of parameters are estimated from these data: brain signatures that characterize brain activity during a state and durations (sojourn times) which specify the probability that states will last different numbers of time points. With these parameters state occupancies can be calculated, which are the probabilities that a time point is in a state. Summing these state occupancies yields estimates of the state sojourn times for each trial

Principle component analysis (PCA) captures correlated sources of variance and the systematic variance common across subjects. The resulting components form an uncorrelated basis set with the first few components capturing the majority of the variance across subjects. The deconvolved data for the 60 runs were concatenated and yielded a 20,747 time‐point × 47,315 voxel matrix. These data were then l2‐normalized, and a spatial PCA was performed. We typically work with the first 20 components and in this experiment the first 20 accounted for 48.9% of the total variance of the deconvolved data.^3^ These 20 components were retained and *z*‐scored to ensure all components are of the same scale. Additionally, this transforms the data such that it can be thought of as roughly distributed as multivariate standard normal (though with heavier tails) for the purposes of likelihood computations used by the method. The result is a 20,747 × 20 matrix. Finally, all time points associated with incorrect trials and trials containing greater than 2 time points that had excessive outliers were removed. The resulting 18,206 × 20 matrix was used in the HSMM‐MVPA analysis. This represented 1,286 trials (654 Verbal problems and 632 Example problems) that varied in length from 9 to 26 time points (points 2 s apart) with a mean length of 14.16 time points. This provides the input to the HSMM‐MVPA method (see Figure 4a).

### 2.4 HSMM‐MVPA

#### 2.4.1 Methodology

The family of models we consider are those that are purely sequential. There is no branching between states: each state of an *N*‐state model has an associated brain signature and sojourn time distribution, and is guaranteed to follow the previous state^4^ (though it is possible to skip states in a sequence). Fitting an HSMM‐MVPA model involves estimating a set of parameters that maximize the likelihood of the *z*‐scored PCA data. The parameter estimation process uses an expectation maximization algorithm (Dempster, Laird, & Rubin, 1977), starting with neutral parameters^5^ and iteratively re‐estimating parameters until convergence. There are two sets of parameters (see Figure 4b)—one set to specify the distribution of state sojourn times and another set to specify the brain signatures.

1. The distribution of sojourn time in each state. The state sojourn time distributions specify the number of time points that a subject spends in a state. We discretize the continuous gamma distribution to obtain a distribution of times that have the skewed property typical of latency distributions. The probability of spending *m* 2‐s time points (a time point accounts for the 2 s it takes to acquire 1 functional brain volume) in state *i* is calculated as:
  $$G\left(m\right|\;v_{i},a_{i})=\int_{2m-1}^{2m+1}\text{gamma}\left(t\right|\;v_{i},a_{i})\mathrm{dt}$$
  where *v*_*i*_ and *a*_*i*_ are the shape and scale parameters for the continuous gamma distribution for the *i*th state.^.^ Note that this means that the probability of spending 0 time points in the state is the probability in the continuous gamma of a duration less than 1 s. In these cases, the state is skipped and the model moves on to a successor of that state.
2. The probability of the fMRI activity in the state. The *z*‐scored PCA components are approximately normally distributed. Their *z*‐scoring allows us to treat them as independent normally distributed values around the mean values for the state with a *SD* of 1. The set *F*_*j*_ of observed component scores *f*_*jk*_ at time j for components *k* will have probability:
  $$P\left(F_{j}\left|M_{i}\right.\right)=\prod_{k=1}^{20}\text{Normal}\left(f_{\mathrm{jk}},\mu_{\mathrm{ik}},1\right)$$
  where *M*_*i*_ is the set of means *μ*_*ik*_ estimated for state *i*.

While the underlying model treats each time point in a trial as in a single state, the estimation process considers all possible ways of partitioning a trial of *M* time points into *N* states.^6^ Let *m*_1_ + *m*_2_ + ⋯ + *m*_*N*_ = *M* be one such partitioning where *m*_*i*_ is the number of time points in state *i*, 1 is the start state, and *N* is the end state. The probability of this interpretation is

$$p\left(m_{1},m_{2},\ldots ,m_{N}\right)=\prod_{i=1}^{N}\left[G\left(m_{i}|v_{i},a_{i}\right)\prod_{j}^{m_{i}}P(F_{j}|M_{i})\right]$$

The estimation process calculates the summed probability of all such partitionings. This is the probability of the data in that trial. HMM algorithms can efficiently calculate the summed probability using dynamic programming techniques (see discussion of explicit duration HMMs in Yu, 2010). The parameter estimation process seeks to minimize the summed log‐likelihood of all trials.

While each partitioning treats a time point as being occupied by a single state, the probability of a state at a time point is the summed probability of all partitionings that assign that state to that time point. This yields the State Occupancies as illustrated in Figure 4c. Summing these probabilities over all time points and multiplying by the 2‐s duration of a time point gives the mean duration of a state on a trial, as illustrated in Figure 4d.

#### 2.4.2 Model selection and fitting

As noted above, the models we are considering are purely sequential. Nonetheless, such models can be somewhat complex. There are two main decisions that need to be made that impact model complexity and accuracy of the resultant model: identification of the appropriate number of states and whether to use an informed or uninformed model specification.

##### Number of states

The search for good model fits is currently done using a leave‐one‐out cross‐validation (LOOCV) procedure. Assuming a model with some number of states *N*, this approach estimates the maximum‐likelihood parameters for all but one of the subjects and then uses these parameters to calculate the likelihood of the data from the last subject. This likelihood measures the success of using these parameters to predict the data of the left‐out subject. The process is rotated through all the subjects and so can calculate the predicted log‐likelihood of the data for each subject assuming the *N* states.

The data of the all‐but‐one subjects should be fit better with more states because there are more parameters, but this is no guarantee that more states will predict the data of the left‐out subject better. If using more states is just overfitting, the predicted likelihoods will not be better. A simple sign test can be used to identify “better” models by noting whether the number of subjects better predicted is more than would be expected by chance. An *N*‐state model is justified if it predicts better significantly more subjects than models with fewer states. More generally, a model with more parameters is to be preferred over a model with fewer parameters only if it fits significantly more subjects.

##### Model specification

While number of states is one salient feature determining model complexity, equally important is the number and types of parameters being fit. Given a particular experiment, we can impose various constraints on brain signatures or latency distributions. In the current study, except for the Study Phase, the two study treatments result in similar times and variability (Figure 3) among the remaining behavioral phases of a trial. Therefore, we chose an HSMM that is free to estimate different timing parameters and brain signatures for the first state but constrained to fit the remaining states with a set of common parameters between the two experimental conditions. To illustrate and assess the consequence of this choice, we also fit two other HSMMs that started with less knowledge of the experiment. The least informed model did not know about the two study conditions and rather fit the same time parameters and brain signatures to all trials. A more informed model knew there were two conditions but did not know about where the condition differences might lie and so estimated separate time parameters and brain signatures for all states. These three models are referred to as State‐1‐Separate, All‐States‐Shared, and All‐States‐Separate. The All‐States‐Shared model had the fewest parameters, estimating just one set for all states. The All‐States‐Separate model had the most parameters, estimating two sets for all states. The State‐1‐Separate model estimated two sets only for the first state.

#### 2.4.3 Evaluation of models

**Figure 5:**
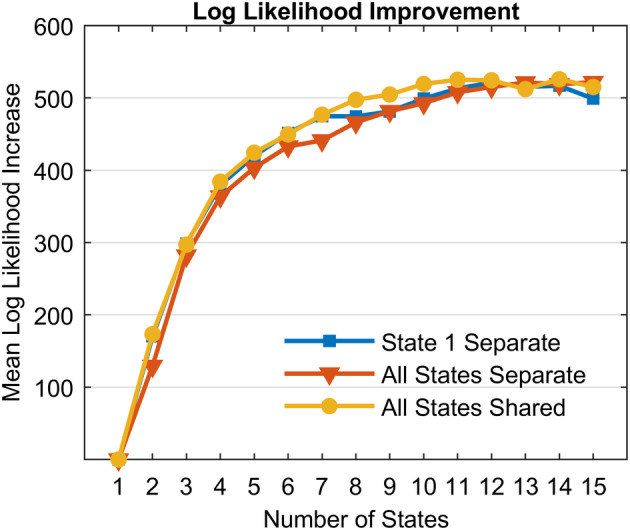
Improvement in mean log‐likelihood over 1‐state models for three types of HSMM‐MVPA model specifications

Figure 5 shows the improvement in mean log‐likelihood of the data for the left‐out subjects during LOOCV as a function of number of states estimated. This is represented as the improvement over just a single state model. The largest effect is driven by number of states rather than the difference among the model choices. Informed and uninformed all converge to similar asymptotic performance around 12 states. The 12‐State State‐1‐Separate model is the best fitting of all models in terms of fitting more subjects better.^7^

**Figure 6:**
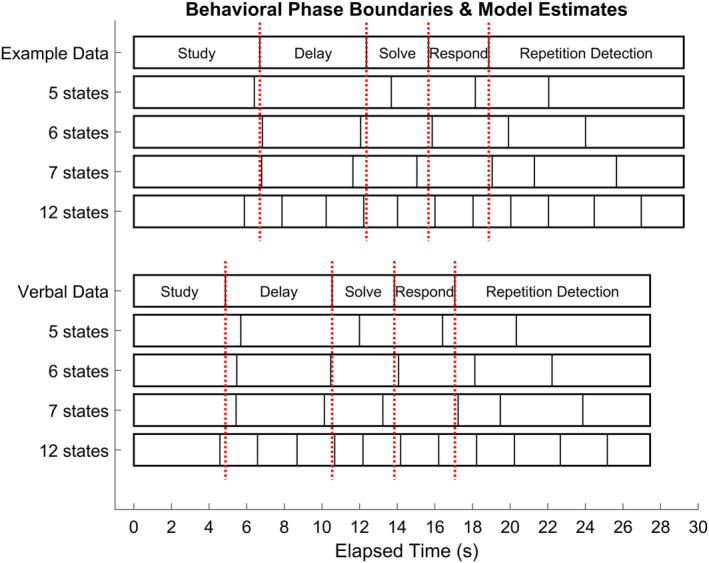
Comparison of mean state sojourn times and mean phase durations for each condition of the experiment. Results are shown for State 1 Separate models of differing numbers of states. Dotted vertical lines denote behavioral phase boundaries

Focusing on the State‐1‐Separate models, the best fitting value of 12 states is many more than the five phases in the experiment. The finding of additional states is not unexpected, as the each of the behavioral phases of the experiment may involve multiple cognitive demands. The question of interest is whether some state boundaries align well with the behavioral task boundaries of the experiment. Figure 6 compares the average durations of the five behavioral phases with the average sojourn times for various numbers of states:

5 States: This is the fewest number of states that could match to the behavioral phases. The boundary between the first two states matches fairly closely the boundary between Study and Delay. However, it has only two states to cover the three phases of Delay, Solve, and Respond and identified two states in the Repetition phase.
6 States: This is the smallest number of states that seems to capture the boundaries between the phases. The extra state again is a division of the repetition phase into two states. The root‐mean‐square deviation (RMSD) between the four phase boundaries and the first four state boundaries is 0.59 s.
7 States: Adding an additional state results in the further division of the repetition‐detection phase into three states. The RMSD between the 4 phase boundaries and the first four state boundaries is .48 s.
12 States: This is the best model as indicated by likelihood improvement in the LOOCV. Again, there are state boundaries near the behavioral phase boundaries, but they have a RMSD of .56 s. Which is not as good as the 7‐state solution. As we will demonstrate with synthetic data, sometimes the best‐performing model in LOOCV can have more states than actually generated the data.

**Figure 7:**
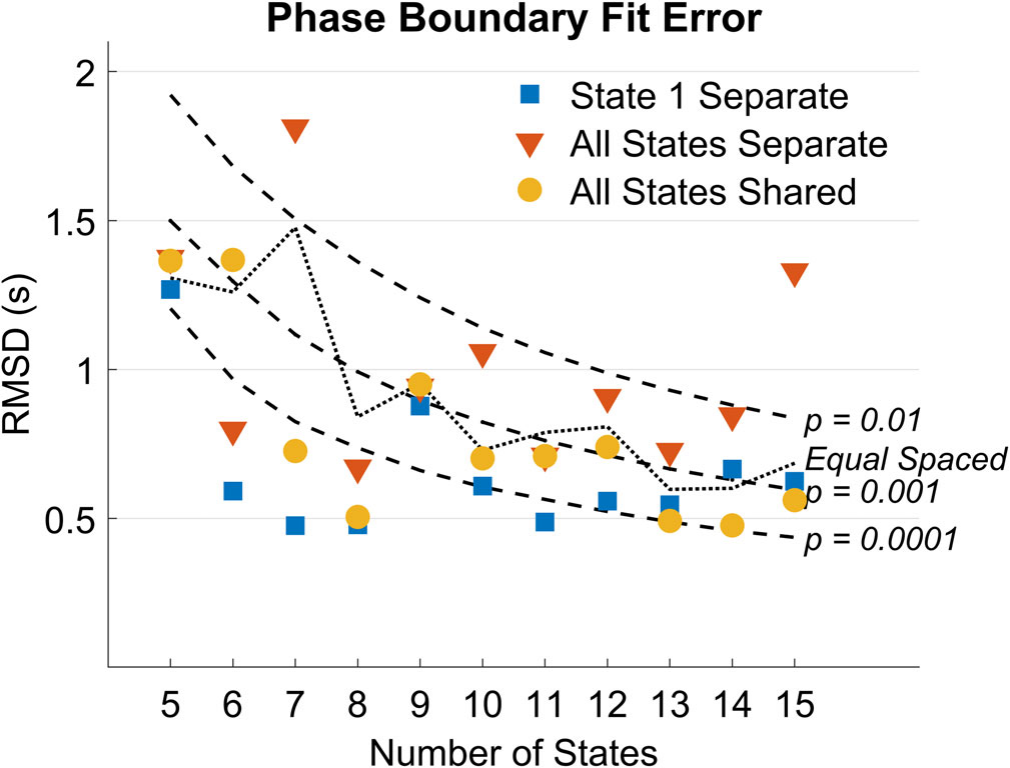
Deviation between behavioral phase boundaries and estimated boundaries from different models shown with several measures of chance performance

Figure 7 shows the RMSD for the three models for different numbers of states and compares this to various measures of chance performance. Chance at *p* = .01, *p* = .001 and *p* = .0001 levels were calculated as follows: For an *N*‐state model *N* − 1 boundaries were placed with equal probability anywhere from the beginning to the end of condition. Then for each of the actual *N* − 1 mean phase boundaries (Figure 6) we calculated its distance from the closest of the randomly placed boundaries and an RMSD for these random boundaries. Repeating this process 10,000,000 times provided a basis for determining how likely a random placement of *N* − 1 boundaries would achieve various measures of RMSD. As another baseline for comparison, the Equal Spaced series in Figure 7 shows chance performance as a function of number of states where the state boundaries are simply chosen to be temporally equally spaced. From 5 to 12 states, the State‐1‐Separate models are doing best at capturing behavioral phase boundaries. The 5–13 state models are doing better than the .001 chance measure and the 6, 7, 8, and 11 state models are doing better than even the .0001 threshold. The 7‐state State‐1‐Separate model is performing best at identifying the behavioral phase boundaries, achieving the same level of fit as the 8‐state model with one fewer state.

**Figure 8:**
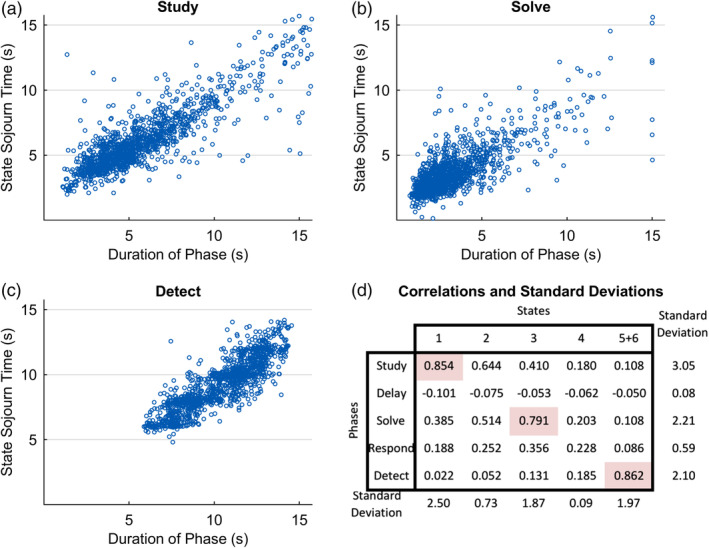
(a)–(c) Correspondence between true behavioral phase durations on a trial and estimates from the State 1 Separate 6‐state model. Best fitting linear equations: (a) *Y* = −0.62 + 1.04*X*; (b) *Y* = −0.17 + 0.94*X*; (c) *Y* = 1.82 + 0.92*X*; (d) correlation between all phase durations and sojourn time estimates

Given that the focus is on behavioral phase boundaries and not within‐phase states, we chose to focus on the 6‐state State‐1‐Separate solution because it is the fewest state solution where predicted boundaries and the five behavioral phase boundaries appear to align. While its average state boundaries are close to the average phase boundaries, it is another question how well it can capture the trial‐by‐trial variation in phase duration. Figure 8a–c shows the correspondence for the three high‐variance phases and the corresponding states (combining States 5 and 6). There is a striking clustering along the main diagonal (in each case the best fitting line has a slope of about 1). Figure 8d gives all intercorrelations between pairs of phases and states with the high variance pairs highlighted. The high correlations are those illustrated in Figure 8a–c. In total, Figure 8 shows that the HSMM‐MVPA method is able to capture with a fair degree of accuracy the duration of individual phases on individual trials.

**Figure 9:**
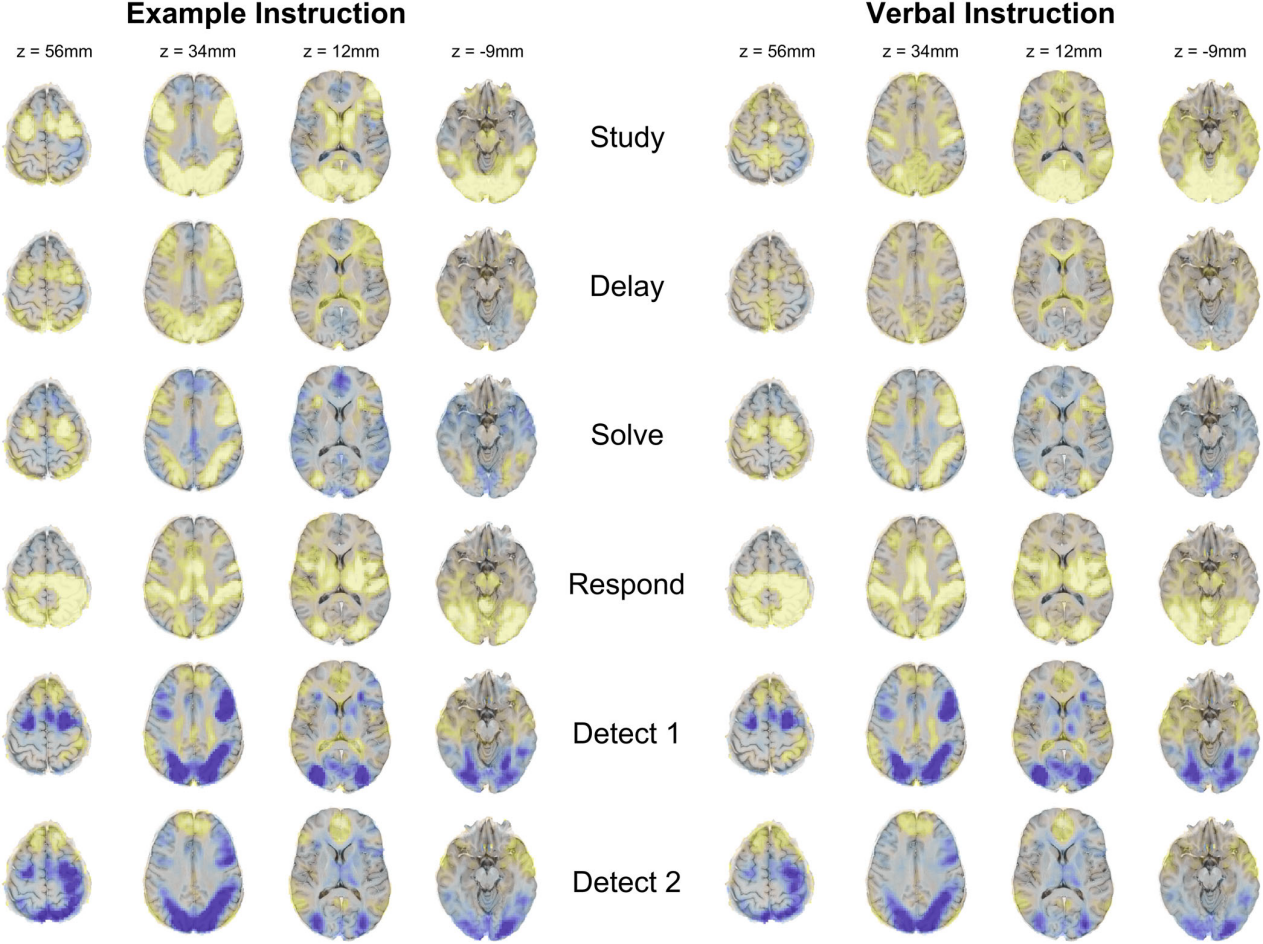
Mean activity during each state for each instruction condition. Darker regions reflect negative activity values and lighter regions reflect positive activity values. The z‐coordinates refer to the location of the midpoint of each slice in Talairach space. Note brain images are displayed following the radiological convention with left brain shown on the right

The analyses so far have been based on the 20 PCA values constructed from 47,315 voxels. Figure 9 goes back to the deconvolved voxel patterns and displays the average activity in each state for each condition.^8^ The activity patterns are quite similar between the two conditions across all corresponding states with the exception of the Study states (RMSD per voxel of .05% for corresponding states compared to .22% for noncorresponding states). The two Study states are slightly more similar than they are to other non‐corresponding states (.15% versus .22%). The Study states show high activity in visual areas in both conditions. When studying an example, there is more prefrontal and parietal activity than when studying verbal instructions. The two Solve states also both show increased parietal and prefrontal activation. The two Respond states show particularly high activity in the motor areas. The Detect states show decreased activity in prefrontal and motor areas. The activity pattern between Detect 1 and Detect 2 are quite similar but Detect 2 shows increased polar frontal activity suggesting subjects are entering default mode.^9^ Thus, the HSMM‐MVPA method has not only found sensible temporal patterns but also sensible spatial patterns in the brain.

Summarizing the results thus far, when considering data from an actual experiment, the HSMM‐MVPA method was able to identify sensible states both in terms of their matchup to actual behavioral phase patterns (Figures 6 and 8) and in terms of the brain activation patterns in the identified states (Figure 9). While we have shown that indeed the method recovers task boundaries accurately and yields sensible activation‐based results, there remain a few open issues that can only be addressed using synthetic data. First, we will use synthetic data that has properties similar to the experimental data to directly assess the inferred model accuracy for both latency and activation patterns. To paint a more general picture, we will examine results over varying levels of signal‐to‐noise. Finally, the remainder of the article examines application to a range of synthetic data generated from different ground‐truth state structures. This will provide information about the kinds of experiments where we might expect accurate and useful models.

## 3 HSMM‐MVPA APPLIED TO SYNTHETIC DATA

Results from the experimental data suggest two main issues that need to be addressed: direct assessment of model derived sojourn times and activity patterns, and determining the best number of states suggested by the LOOCV procedure. With the experimental data, evaluation of model goodness was assessed on how well task phase boundaries aligned with model derived boundaries, and the reasonableness of the state activity patterns. In this section, we transition from the 6‐phase experimental data and focus on simulated data with a 6‐state ground‐truth. Doing so allows us to directly assess the goodness of discovered models by measuring how well models can capture the now known ground truth state sojourn times and activity patterns. We then consider more general 6‐state models, and finally the determination of numbers of states.

### 3.1 Direct assessment 1: Synthetic experimental data

Building on the experiment results, the next set of analyses uses the 6‐state State‐1‐Separate model from Section 2 as the ground truth, generating synthetic data sets using those activity patterns and sojourn times as ground truth. Note in the case of the real experimental data, there was not ground truth as to the true number of states, but only the knowledge that some of the state boundaries should align with the behavioral phase boundaries. Here the goal is to understand how well the HSMM‐MVPA technique performs in recovering known ground truth states, and how well it does so across varying amounts of noise.

The HSMM‐MVPA method model assumes a distribution of activity patterns in a state and distributions of state sojourn times. This first set of synthetic simulations used the state sojourn times that were estimated for each of the 1,286 real trials. This provides temporal distributions of state sojourn times that reflect both within‐subject and between‐subject variation. The synthetic data for each time point of each trial were then generated as a weighted combination of noise and signal appropriate to the states for those time points:

1. Signal: The signal was based the six brain signatures estimated in the experiment. To incorporate individual differences in brain activity, brain signatures were created for each subject that were an equal weighting of that subject's mean activity during each state and the grand average state signatures.^10^ The signal for a time point *t*, *S*(*t*), was a combination of the brain signatures, *B*_*i*_, of the states *i* weighted by how much of the time point the states occupied, *w*_*i*_(*t*)

$$S\left(t\right)=\sum w_{i}\left(t\right)B_{i}$$

65% of time points were occupied by a single state, in which case the signal was just the brain signature of that state.

2. Noise. There were two important properties of the data to capture in the noise that is added to the signal. First, the *z*‐scored PCA scores have more extreme values than would be expected from a standard normal distribution—0.39% of the scores are greater than 4 *SD*s from the mean, which is about 60 times more than would be expected with a normal distribution. Second, there is a temporal correlation between adjacent measures. The values in one time‐point have a correlation of .77 with the values in the next time point, .34 with the values in time points that are 2 time points downstream, and .08 with time points that are three time points downstream. Data that approximate these properties can be created by making the noise for the sample *t*, *N*(*t*), a running average of four samples from a *t*‐distributions with degrees of freedom 3, *T*_3_:

$$N\left(t\right)=T_{3}\left(t\right)+T_{3}\left(t+1\right)+T_{3}\left(t+2\right)+T_{3}\left(t+3\right)$$

The *t*‐distribution provides the excess of extreme values. Temporal correlation is created because adjacent noise samples share some t samples in their sum.

The synthetic data, *F*(*t*), is created as a weighted sum

$$F\left(t\right)=S\left(t\right)+a_{\mathrm{SNR}}*N\left(t\right),\;a_{\mathrm{SNR}}=\text{sqrt}\left[\sigma^{2}\left(S\right)/\left(\sigma^{2}\left(N\right)*\mathrm{SNR}\right)\right]$$

where SNR is the chosen signal‐to‐noise ratio. These values are then *z*‐scored and have extreme values truncated to the range −5 to 5 just as the actual data was processed described earlier. This generates a matrix of 18,206 time points × 20 dimensions for the synthetic data like the original data with similar properties. Models can then be fit to the resulting data.

**Figure 10:**
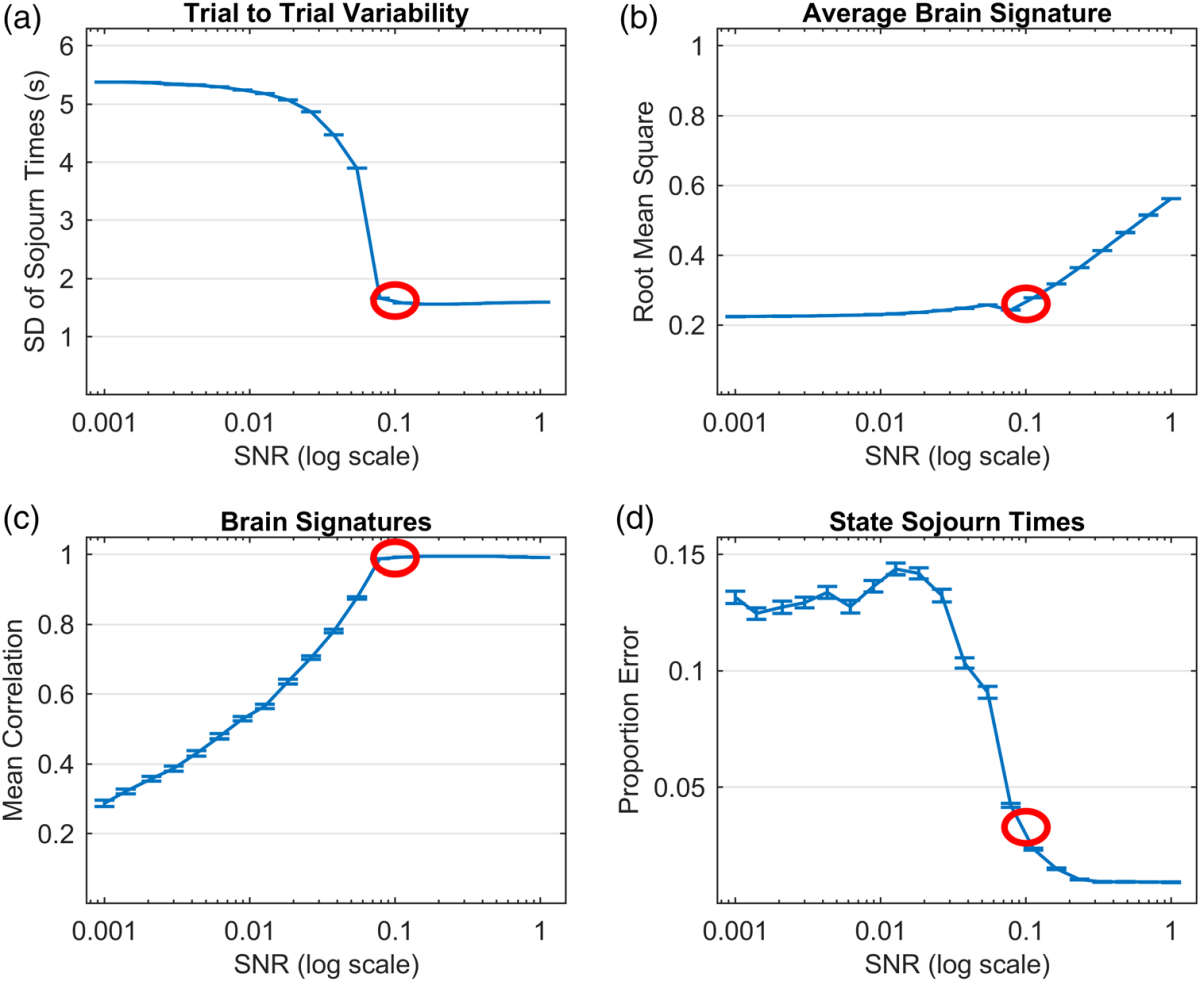
(a) Measure of the variability in state sojourn times; (b) measure of the magnitude of the brain signature; (c) correlation between estimated and true brain signatures; (d) accuracy in estimated state sojourn times. Circles highlight values at SNR of .1 that characterize the Lee et al. experiment

We fit the 6‐state model to synthetic data generated with different signal‐to‐noise ratios from .001 to 1.0. Figure 10 shows the effect of SNR on various properties of the resultant model estimates. Figure 10a,b provide measures that point to a value of SNR that characterizes our experiment. Figure 10a shows how the trial‐to‐trial variability in estimated state sojourn times decreases with increasing SNR. It drops off sharply from a SNR of .01 to .1, reaching an asymptotic value close to the 1.52 trial‐to‐trial variability in state sojourn time that we estimate, Figure 10b shows how the computed root‐mean‐square (RMS) of the estimated brain signatures^11^ increases with signal‐to‐noise ratio. The RMS of the brain signatures increases sharply after a SNR of .1, reflecting how the signal becomes dominated by the brain signatures. The RMS value we obtained for our brain signatures was .265. The circles in Figure 10a,b show the values for the experiment (the *SD* in sojourn times was 1.52 and RMS of the brain signatures was .265). The correspondence between these measures for synthetic and experiment data suggest that 0.1 is a reasonable estimate of SNR of the experiment data. The synthetic data at this setting has similar properties to the real data: proportion of values greater than 4 *SD*s from the mean (.34%) and correlations of brain signatures at various lags (.75, .28, and .22 for lags 1, 2, and 3, respectively).

Parts (c) and (d) of Figure 10 show measures of how well the HSMM‐MVPA method can recover the ground truth (these are measures that will be reported for other synthetic data sets as well). Figure 10c shows how well the estimated brain signatures correlate with the generating brain signatures as a function of SNR. Figure 10d shows the accuracy in estimating the ground truth state sojourn times, calculated as error in estimate divided by true sojourn time. In both, there is a fairly large loss as SNR drops from .1 to .01. There is good success at recovering ground truth for a SNR of 0.1, which matches properties of the fit to the original data (Figure 10a,b).

### 3.2 Direct assessment 2: General 6‐state model applied to 6‐state ground truth

The synthetic data used in Section 3.1 were a rather special case of ground truth, both because they were based on the solution of an HSMM‐MVPA and because they reflected the peculiarities of a particular experiment. To get a sense of more general performance, we generated 100 ground truths that were single‐condition^12^ experiments with 6 states. The sojourn times and brain signatures for these states were loosely in the same range as the experiment and reflected the potential of individual differences:

State sojourn times: Each state had a mean sojourn time, *M*_*i*_, selected from a uniform distribution between 2 and 8 s:

$$M_{i}\sim U\left(2,8\right)$$

Six such state sojourn times sum on average to 30 s, which is about the duration of trials in our experiment. However, various ground truth models varied from 20 to 40 s in the summed mean sojourn times of their 6 states. The individual trials were generated from gamma distributions with a shape parameter, a_i_, selected uniformly from 2 to 5 (the range in the fits for the experiment)

$$a_{i}\sim \text{Uniform}\left(2,5\right)$$

The scale parameter of the gamma would then be

$$b_{i}=M_{i}/a_{i}$$

This produced state sojourn time parameters like those estimated for the experiment. To reflect individual differences, the scale parameter for an individual subject j was itself distributed around the mean scale according to a gamma:

$$b_{\mathrm{ij}}\sim \text{Gamma}\left(15,b_{i}/15\right)$$

As a consequence, subject mean state sojourn times varied around population mean state sojourn times with a *SD* of .25 of the population mean sojourn time. The times for state i and subject j would then be gamma distributed on individual trials:

$$t_{\mathrm{ij}}\sim \text{Gamma}\left(a_{i},b_{\mathrm{ij}}\right)$$

Brain signatures: Brain signatures were generated as 6 states of 20 values randomly selected from standard normal.

$$B_{\mathrm{ki}}\sim \text{Normal}\left(0,1\right)$$

which gives the *k*th value for the *i*th state. Subject *j*'s brain signatures were generated by adding to the group signatures values another set of standard normal weighted .5.

$$B_{\mathrm{kij}}\sim B_{\mathrm{ki}}+.5*\text{Normal}\left(0,1\right)$$

As before, the signal values for individual time points were generated according to the state occupancy of that time point. This was mixed with normal noise according to different SNRs. We considered a reduced range of SNRs—from .009 to .23, which was where the major changes were in Figure 10. To have a similar power experiment as the actual experiment, the synthetic data involved 20 subjects each with 100 trials.

**Figure 11:**
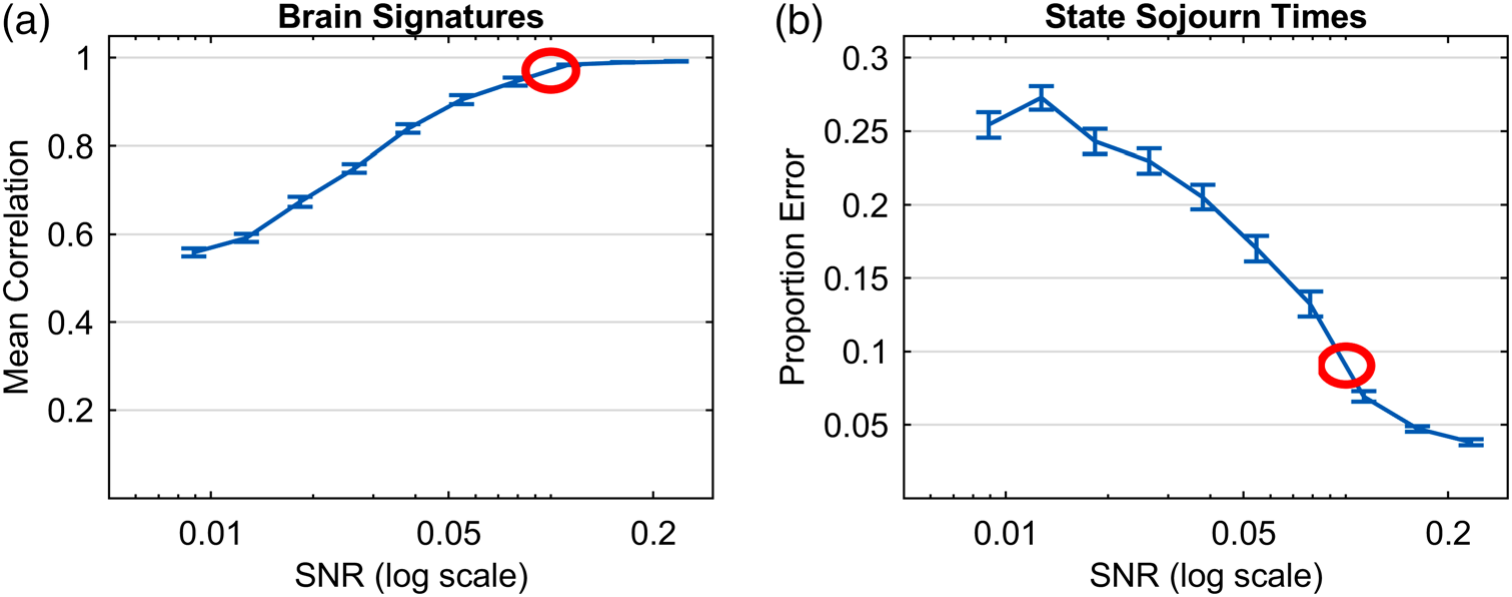
Accuracy in recovering the parameters of an arbitrary 6‐state ground truth generating model for different SNRs. Circles highlight values at SNR of .1 that characterize the Lee et al. experiment

Averaging over the 100 experiments at each SNR, Figure 11a shows the correlations of the generating brain signatures with the estimated brain signatures. Figure 11b shows the error in estimating the state sojourn times calculated as absolute error divided by true sojourn time of that state. While performance is not as good as the special case in Figure 10, Figure 11 shows relatively good performance with SNRs greater than .05. At a SNR of .1, which seemed to characterize our experiment, the average correlation of the estimated brain signatures with the ground truth signatures is about .98 and the average error in estimating state sojourn time is about 8% of the true sojourn time.

### 3.3 Assessing number of states found by LOOCV

Figure 11 illustrates fits of 6‐state models to data where the ground truth is 6 states. Recall, however, that in the experimental data (Section 2), models with greater than 6 states were discovered, with the maximal‐likelihood model having 12‐states. While the 12‐state model did well capturing experiment phase boundaries, it did not do quite as well as fewer‐state models. Here, we consider the issue of models that LOOCV has identified as best in terms of maximal likelihood, yet that have greater than the six ground‐truth states. In order to understand how such models are to be interpreted and the conditions of the data that can result in solutions with greater than ground truth numbers of states, we simulated 4 sets of 50 datasets, each under different constraints. All datasets were generated similarly to those of the prior section and reported in Figure 11 but with the signal‐to‐noise ratio fixed at 0.1. The two factors we varied were features that we thought might produce more states than are in the ground truth. First, the temporal correlation in the noise might mean the procedure over‐estimates the likelihood of excess states. Second, each partitioning assumes a whole scan is occupied by a state, even though averaging over partitions produces the fractional state occupancy (Figure 4d). Extra states might be created to handle scans that are mixes of adjacent states. Therefore, the two factors we varied were whether the added noise was temporally correlated or not, and whether the 6 ground‐truth states partially or wholly occupied the 2‐s time‐points that make up individual trials.

**Figure 12:**
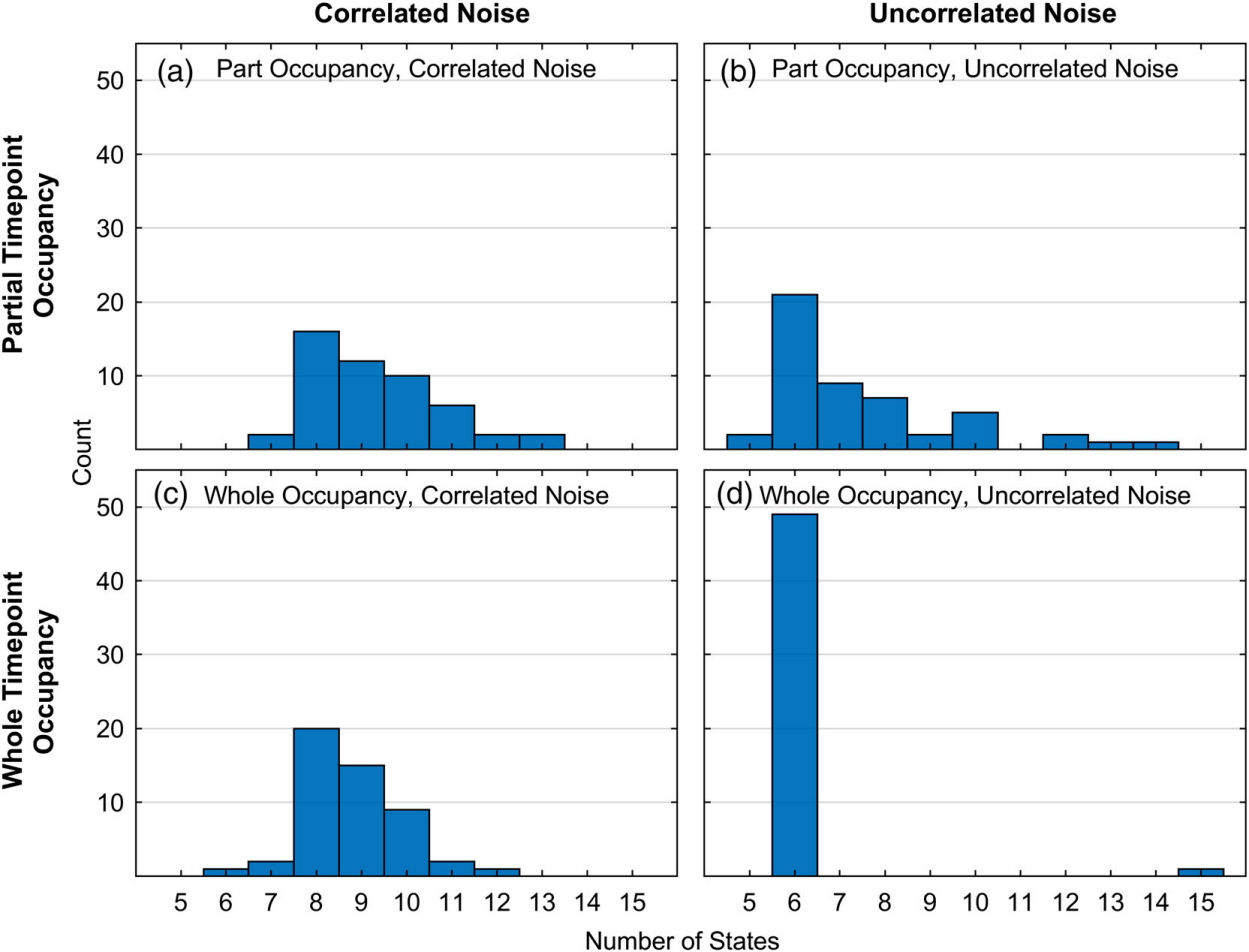
Number of 50 data sets (generated from a ground truth of 6 states and SNR of .1) best fit by models of differing numbers of states. The four panels reflect whether there was correlated noise in the data sets and whether ground truth states could occupy a fraction of a time point

Figure 12a summarizes the maximal‐likelihood number of states gotten from LOOCV for each of the 50 datasets generated with temporally correlated noise where ground‐truth states were allowed to partially occupy 2‐s time‐points. The simulated data in this cell reflect the same construction as that described for simulation results shown in Figure 11. Using a threshold of 16 subjects predicted better (*p* < .01 under a sign test), each of the 50 synthetic data sets were better fit by models with 7–13 states, the mode being an 8‐state solution (32% of the cases).

Examining the brain signatures estimated for the best state solutions selected by the LOOCV method, each of the six generating brain signatures was correlated with one or more of the estimated brain signatures. Extra states had brain signatures related to the original 6 in two ways:

Splits: In some cases, the better fitting model had two states corresponding to one of the generating states. The brain signature for the first state was also correlated somewhat with the brain signature for the preceding state while the brain signature for the second state was correlated somewhat with the subsequent state.
Bridges: In some cases, a state typically 1 time point in length, was placed in between two generating states. This state correlated relatively strongly with both of the adjacent states. This state also captured the transition between the two states.

The HSMM‐MVPA method assumes that whole states occupy time points even as it considers all ways of partitioning states into sequences of time points. Some time‐points will have a mixture of two states and better fits can sometimes be obtained if a state is generated that is a blend of two states. Further, adjacent states include temporally correlated noise as well, also contributing to the need for bridge states. The remainder of Figure 12 shows results from the rest of the simulated data, illustrating the relative contributions of temporally correlated noise and partial occupancy of time‐points to discovery of solutions with more states than ground truth.

Figure 12b shows results for each of the 50 datasets generated where the noise was not temporally correlated, but where ground‐truth states were still allowed to partially occupy 2‐s time‐points. When correlated noise is not present, we are less likely to find better higher‐state solutions with the mode at 42% 6‐state solutions matching ground truth. Figure 12c shows results for the 50 simulated datasets with temporally correlated noise, but where 2‐s time points were wholly occupied. Similar to the partial occupancy results in Figure 12a, 98% of the solutions were greater than six states, with a mode of 40% 8‐state solutions being best. Finally, Figure 12d shows results for 50 simulated datasets generated with uncorrelated noise and whole time‐point state occupancy. For these datasets, one can see that there is little need of bridge states. This is reflected in the results: 98% of the best solutions are in fact 6‐state solutions.

In almost all cases, the 6 state solutions were better than fewer states and corresponded closely to the ground truth. However, because of the fact that there is temporally correlated noise and states partially occupy time points, the LOOCV may well select a higher‐state solution as better. When this happens, adjacent states in the higher‐state solution will be correlated. This indicates that one should be cautious about selecting high state solutions where adjacent states are correlated. In past research we have chosen not to use a higher‐state solution when there were correlated states. For instance, Anderson, Pyke, & Fincham (2016) and Anderson, Zhang, et al. (2016) used a 4‐state solution even though LOOCV preferred 5 states with 2 correlated states.

### 3.4 Exploration of more general ground truth state structures

#### 3.4.1 State recovery as a function of number and sojourn times of ground truth states

**Figure 13:**
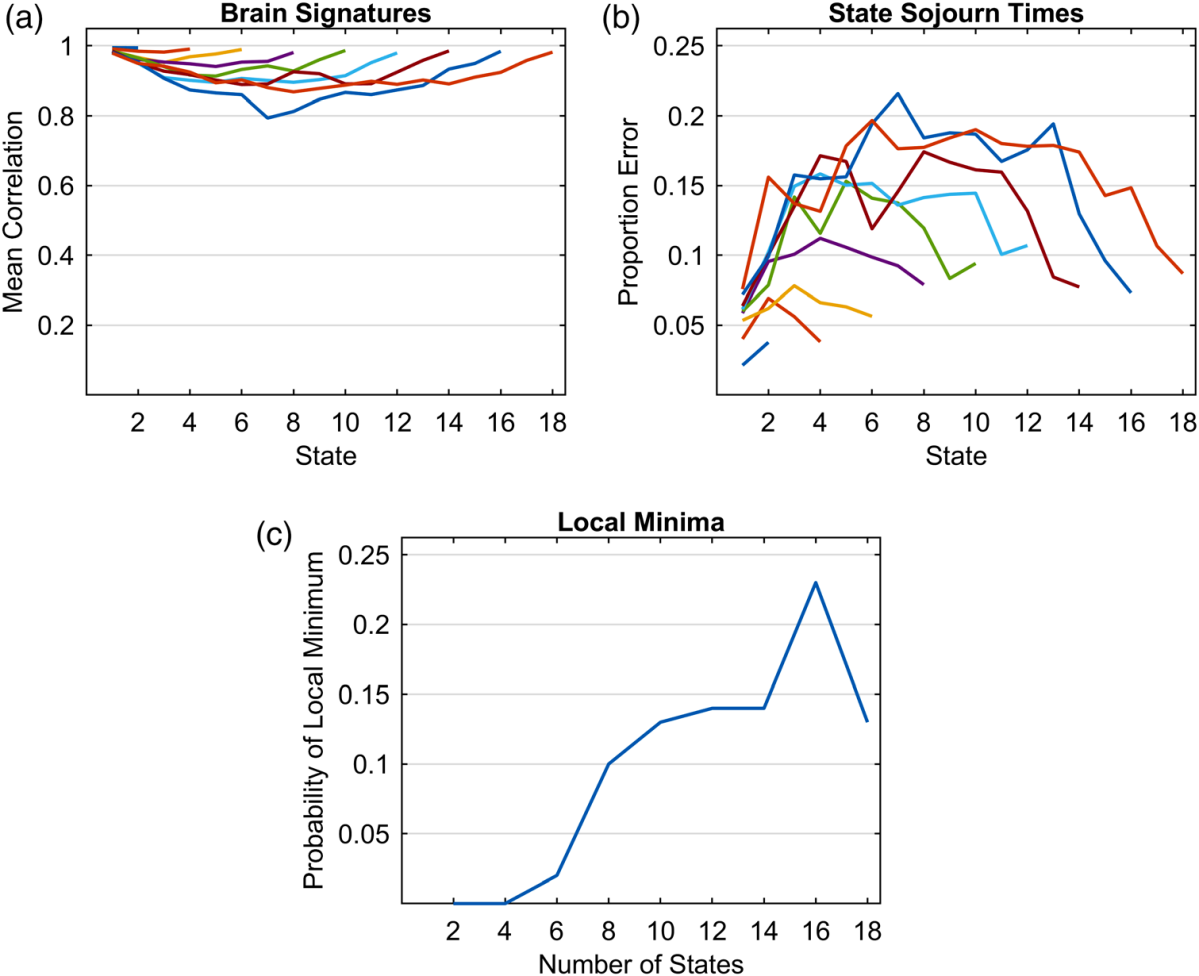
Recovery of ground truth generating models with different numbers of states: (a) Correlations of recovered brain signatures with generating brain signatures; (b) mean proportion error in estimating state sojourn times; (c) proportion (out of 100 synthetic data sets) of convergence to local minima from neutral parameters. SNR = 0.1

Results thus far show fairly successful recovery of 6‐state structures with a SNR of .1. To explore success with different numbers of states, we generated cases where the ground truth varied from 2 to 18 states, looking only at the cases of an even number of states. The brain signatures and sojourn times for the states were randomly generated as in the previous simulations using again 20 subjects with 100 trials each. For each choice of states, 100 data sets were generated with a SNR of .1. Figure 13a shows the ability to recover the generating brain signatures and Figure 13b shows the accuracy in recovering state sojourn times. Basically, the accuracy at recovery is high for the beginning and end states and decreases as the states are further from these end points. The brain signature correlation for the middle states of the large number of states is about .85 and proportion error is about .18 of true means. These are fairly good but Figure 13c adds a cautionary note. It shows how often local minima fits were obtained when starting from neutral parameters. While this is infrequent with small numbers of states, it rises to about 20% as the number of states increases.^13^ The local minima fits typically do a very poor job of recovering the state structure of the experiment. The results shown in Parts (a) and (b) would be considerably worse for high numbers of states had the local minima fits been used.

**Figure 14:**
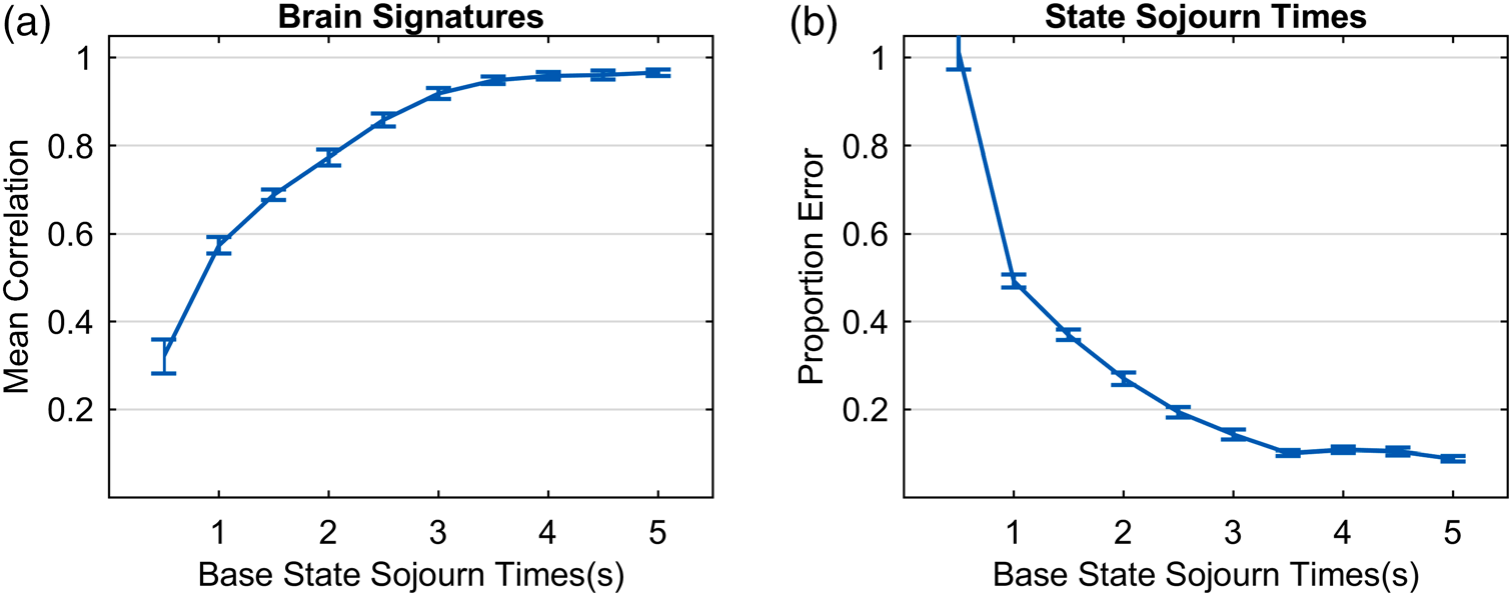
Accuracy in recovering the parameters of 6‐state ground truth generating models with different mean state sojourn times: (a) Correlations of recovered brain signatures with generating brain signatures; (b) mean proportion error in estimating state sojourn times. SNR = 0.1

Figure 13 identifies limitations that arise with more states. What about effects of the brevity of states? So far, we have considered cases where states averaged between 2 and 8 s. To explore success in recovering short states, we varied the base sojourn time of a state from .5 to 5 s keeping the number of states at 6. Mean sojourn times of individual states in a synthetic state structure could vary from .4 to 1.6 the base sojourn time.^14^ As in the previous cases, 100 data sets were generated for each duration with a SNR of .1 (20 subjects, 100 observations per subject). Figure 14a shows the ability to recover the generating brain signatures and Figure 14b shows the accuracy in recovering state sojourn times. Accuracy at recovery of state structure for the shortest base sojourn time of .5 s is particularly bad and accuracy increases close to an asymptote at about 3.5 s. Part of the limitation at short sojourn times may reflect the use of 2 s time points but the slow temporal properties of the BOLD response and the autocorrelation in the signal are likely also important in limiting accuracy for brief states.

#### 3.4.2 State recovery with multiple conditions

**Figure 15:**
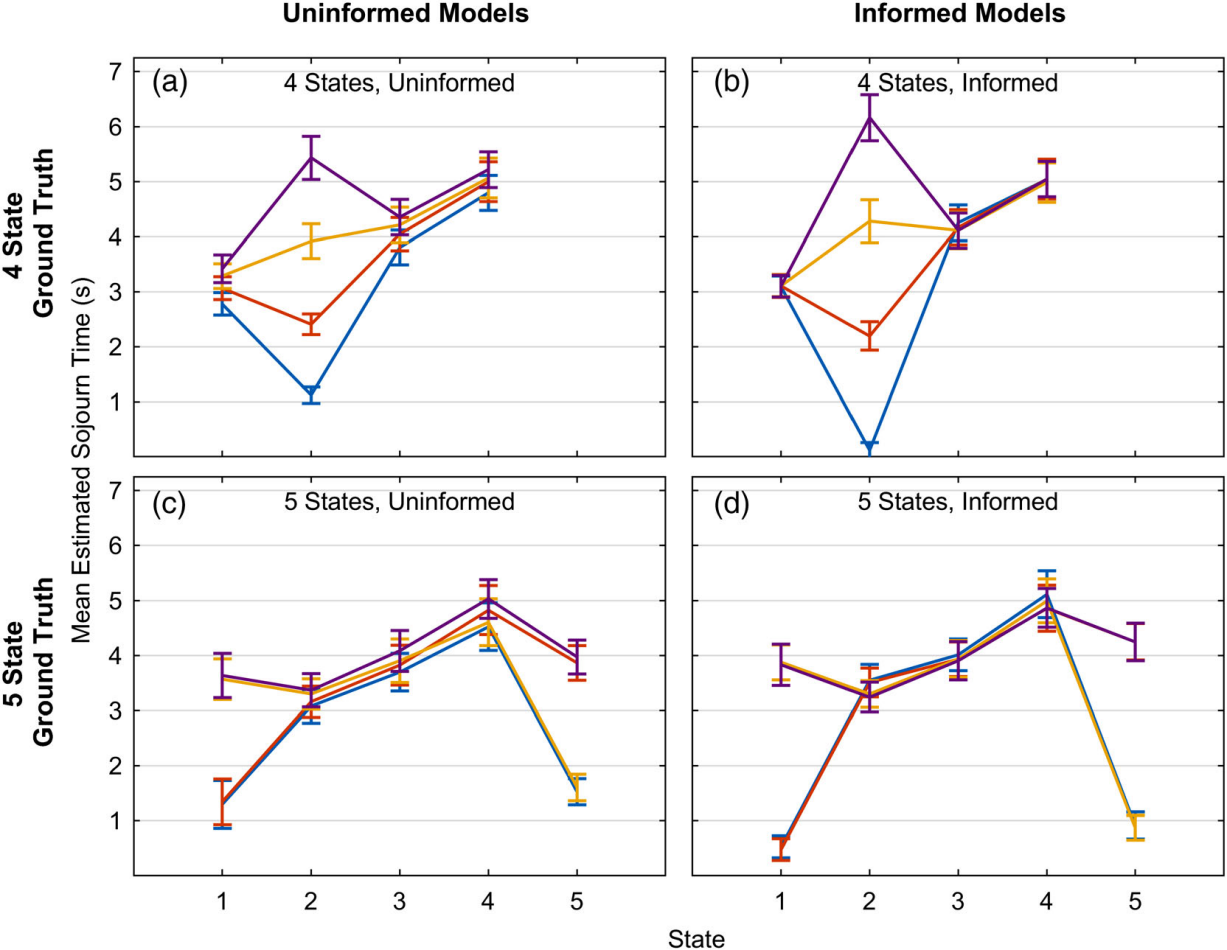
Recovered state sojourn times for ground truth generating models where some state sojourn times vary with condition: (a) and (b) 4 states where state 2 varies; (c) and (d) 5 states where states 1 and 5 vary. SNR = .1

Except for the simulations of the data from the real experiment, both data and models have just had a single condition. We simulated two experiments where the sojourn times of some states varied as a function of 4 conditions. Past experiments (e.g., Anderson & Fincham, 2014; Anderson et al., 2015; Anderson, Pyke, & Fincham, 2016; Anderson, Zhang, et al., 2016), where we have used HSMM‐MVPA, have had multiple conditions and we were interested in what states these conditions affect. Again, 100 data sets were generated with a SNR of .1 and the results for these two models are shown in Figure 15:

Top panels: There were 4 states and the mean sojourn times of states 1, 3, and 4 did not vary with conditions and were 3, 4, and 5 s, respectively. State 2 lasted an average of .5, 2.5, 4.5, and 6.5 in the four conditions.
Bottom panels: There were 5 states and the mean sojourn times of states 2, 3, and 4 did not vary with conditions and were 3, 4, and 5 s, respectively. States 1 and 5 either were 1 or 4 s. Crossing these possibilities yielded the four conditions.

Figure 15 illustrates the fit of two types of models to each experiment. The less informed models (Figure 15a,c) fit a single model with the correct number of states to all the data and then calculated the mean sojourn time of the states for the trials in each condition. This reflects one approach to discovering what states are affected by condition. The more informed models (Figure 15b,d) fit a model that estimated separate sojourn times for the states that varied with condition^15^ while estimating the same sojourn times for other states. This reflects a situation where one knows what state is affected and wants to estimate this effect. The panels in Figure 15 show the mean sojourn times of the states for the trials for each condition. The HSMM‐MVPA does a good job in all cases of identifying the sojourn times of the states. The error bars reflect 1 *SD* of the means over 100 simulated data sets. The less informed models tended to underestimate the effect of condition and assign a little of the effect of conditions to other states. The more informed models performed a bit better.

## 4 CONCLUSIONS

We have developed the HSMM‐MVPA methodology on the assumption that the resultant models will accurately identify brain states and their sojourn times, allowing for meaningful mappings between brain activity and corresponding cognitive processes. The critical output of the process is a parsing of a long trial into a sequence of periods of relative constant mental activity. One can then examine the patterns of brain activity during these periods as well as determine how the sojourn times of these states vary with experimental conditions. This article has been concerned with assessing how well the method does at identifying the states that are driving systematic brain activity over the course of a trial and the factors that affect these results. While much of the presentation was concerned with using synthetic data to identify where the HSMM‐MVPA method gave good results, we started out by applying the approach to an experiment that had ground truth about where some of the state boundaries should lie. That ground truth was provided by the five phases of the experiment. The boundaries between these phases were identified fairly well by any HSMM‐MVPA that had 6 or more states. Using a LOOCV criterion, there was evidence for up to 12 states (Figure 5). However, models with fewer than 12 states resulted in better identification of state boundaries (Figure 7).

The best‐performing model in terms of the results of an LOOCV can sometimes have more states than actually generated the data, as shown with synthetic data (Figure 12). This happens in part because actual states do not start and end at the boundaries of the 2‐s time points and because of temporally correlated noise. Fit, measured as the likelihood of the observed time point values, can be improved by creating extra states that are merges of adjacent states. A sign that a model has more states than the state structure that generated the data is high correlation between adjacent states. The choice about number of states should be motivated by theoretical conditions as well as the results of the LOOCV. Certainly, one wants the chosen model to result in better prediction of the imaging data than a model with fewer states. But if a theoretically motivated model satisfies this necessary condition and if states in more complex models are correlated with the states of that model, one should consider working with the theoretically motivated number of states.

If one has a firm idea of the number of states that should be present, the question of interest is whether one can accurately identify them in the imaging data. While there is never a guarantee that the peculiarities of one's imaging experiment will not lead to failure, the results were fairly good for SNRs greater than .05. The noise contributing to the SNR will be determined by quality of scanning and factors of experimental control. The signal contributing to the SNR will be determined by how strongly the individual states differ in their brain signatures.

State recovery decreases somewhat as one seeks to identify more states (Figure 13) and falls of quite sharply for states briefer than 2 s (Figure 14). There was success (Figure 15) at identifying states that were brief as half a second in one condition as long as there were other conditions where the states were longer, enabling a good estimate of the state brain signature.

Many cognitive tasks studied by fMRI average less than 2 s and this methodology is not appropriate for these. We have developed complementary HSMM‐MVPA methods with EEG, MEG and ECoG that have shown success in parsing such brief tasks into states (e.g., Anderson, Pyke, & Fincham, 2016; Anderson, Zhang, et al., 2016; Anderson et al., 2018; Zhang, Walsh, & Anderson, 2017, Zhang, van Vugt, Borst, & Anderson, 2018). On the other hand, the current results indicate that one can be fairly optimistic analyzing fMRI data from tasks in the 5 s to 1‐min range, provided one is not trying to identify a great many brief states. This is where we have had success applying the method to study mathematical problem solving (e.g., Anderson et al., 2014; Anderson, Pyke, & Fincham, 2016; Anderson, Zhang, et al., 2016). More generally, one might expect success in problem solving and reasoning tasks that typically fall within that time range. These are important tasks in understanding complex human cognition and have been relatively neglected in neural imaging. We hope researchers interested in such tasks will find these methods of use. In using these methods, researchers should keep in mind that the method may split single cognitive states into multiple states (Figure 12) and theoretical considerations might motivate choosing a smaller number of states.

### 4.1 Analyses and models

The analyses and models in this article can be obtained at http://actr.psy.cmu.edu/?post_type=publications&p=31445.

## CONFLICT OF INTEREST

The authors declare they have no conflict of interest.

## Figures and Tables

**Figure A1:**
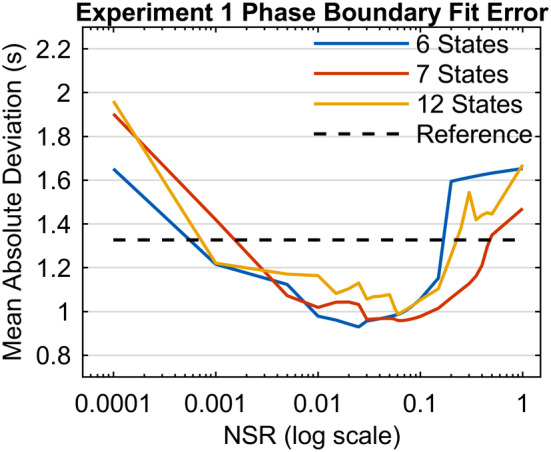
Experiment phase boundary estimate error for 6, 7, and 12 state solutions as a function of NSR used in Weiner filtering during the preprocessing of imaging data. The dashed line Reference shows the smallest possible prediction error when using behavioral data only

## Data Availability

Data sharing is not applicable to this article as no new data were created or analyzed in this study.

## Endnotes

1 The Study and Solving phases by themselves were too brief for useful HSMM‐MVPA results (as we will show). However, the full experimental trial is of the length where a HSMM‐MVPA is appropriate.

2 The last trial of each scanning run is excluded as the deconvolution process to be described results in the tail end of that trial being truncated. Trials having excessive outliers, defined as containing two of more scans where greater than 5% of the voxels were marked as outliers during initial image processing described below.

3 The next 20 accounted for 6.5%. So by 20 dimensions we have reached the point of very diminishing returns in accounting for variance.

4 Note a strict transition order does not necessarily imply that every inferred state will have a unique brain signature. It is possible that a brain signature associated with state k in a sequence of states repeats in one (or more) of states *k* + 2 or later (it likely would not repeat in state *k* + 1 as that case would typically result in the state getting absorbed into state *k* with a longer sojourn time).

5 The neutral starting parameters for a brain signature are 0's for each dimension, the average value over all scans. The neutral starting gamma distribution has a shape of 1 (making it an exponential distribution) and a scale equal to the maximum length of a trial divided by the number of states.

6 The number of such partitionings is (*M* + *N*‐1)!/[*M*! * (*N* − 1)!].

7 Of the other State‐1‐Separate models, it fits 17 or more of the 20 subjects better except for 13 states (14 subjects better) and 14 states (12 subjects better). It fits 17 or more subjects better than the All‐States‐Shared models up to 12 states and 13, 16, and 13 subjects better than All‐States‐Shared models with 13–15 states. It fits 15 or more subjects better than the All‐States‐Separate models up to 9 states and 12, 11, 11, 13, 13, and 13 subjects better than All‐States‐Separate models with 10–15 states.

8 We could use the estimated means for each condition and state to simply reconstruct estimated activity patterns. The result would be identical maps between both conditions for every state except the first. These need not be the case using the procedure that generates Figure 9.

9 Detect 1 has low temporal variability (*SD* across trials is .17 s) while the Detect 2 has relatively high variability (1.88 s). It appears that subjects spend a relatively constant initial period on every trial in the Detect 1 and then move into a more Default‐like mode of processing for the rest of variable phase (which varies in length from 6 to 12 s, Figure 1d).

10 Subjects' brain signatures will deviate from the grand average because of both individual differences and noise. Thus, a representative reflection of true subject is approximately captured by an equal weighting of subject state means and the overall state means.

11 This is derived from the variability of 20 (values) × 6 (states) × 2 (conditions) PCA values that define the brain signatures. The average scan‐to‐scan variability of the PCA scores is normalized to be 1. Thus, if there were no noise the root mean square of the brain signatures would be approximately 1.

12 Later we will examine random multicondition experiments.

13 We discovered that these were local minima by comparing the neutral starting parameter fits with what fits using starting parameters based on the ground truth generation. Without that ground truth generation we would have had to explore a range of alternative starting points.

14 Thus, if the base duration was .5 s individual states could have means from .2 to .8 s. This is the same factor of 4 range as used for the previous synthetic data sets.

15 Unlike the fit to the real data, this model estimated a single brain signature for all states. This is a more common assumption (e.g., Anderson & Fincham, 2014; Anderson, Pyke, & Fincham, 2016; Anderson, Zhang, et al., 2016) where we assume the processing in a state involves the same brain regions in all conditions but might vary in duration with condition.

## APPENDIX 1

As discussed in the main body of the article, through a series of processing steps, voxel time series data are transformed into a form suitable for HSMM‐MVPA processing. One critical step in this processing stream is to generate an estimate of the underlying “activity signal” the drives the measured BOLD response. Time series data from each scanning run are deconvolved using a Weiner filter (Glover, 1999) with a hemodynamic response point‐spread function to accomplish this goal. The hemodynamic response function is the SPM difference of gammas (Friston et al., 2011: gamma(6,1)‐gamma(16,1)/6). The Weiner filter requires an additional parameter that reflects an estimate of the noise‐power‐to‐signal (NSR) ratio in the data. In this article and in other application we have used an NSR parameter of 0.1. Here we analyze the consequences of different choices.

The NSR parameter reflects noise introduced by the measurement of the BOLD signal (the scanner) and subsequent processing steps, and not noise associated with the underlying activity process that yields the BOLD response. There can be a number of possibilities for estimating what might be a reasonable starting point for NSR (e.g., Welvaert & Rosseel, 2013). One possibility is the ratio of variances between a white matter region and a highly responsive brain region. For example, in the current dataset, the ratio of variance of the BOLD signal in a predefined PSPL region to the variance of a predefined white matter region is 0.05. However, it is not clear what the best estimate might be. Here we show that results gotten from an HSMM‐MVPA analysis are quite robust over a fairly wide range of values for the NSR parameter that includes an NSR of .1, used in the article.

### Parameter exploration

We explored a range of NSR for the Weiner filter from .0001 to 1. For each value of NSR, we recomputed Step 2 of the data processing stream described in the main text, followed by the PCA processing in Step 3. The HSMM‐MVPA procedure was applied to each resultant dataset. The measure of performance reported here aligns with evaluation metrics described in the main text: how well we could predict behavioral task boundaries on a trial‐by‐trial basis. For each NSR, we computed the mean absolute deviations (MADs) between predicted and actual behavioral task boundaries.

Figure A1 shows these results for three numbers of states: the 6‐state solution which was minimal state solution that performed well at predicting task boundaries, the 7‐state solution which was best in terms of prediction error, and the 12‐state solution which was best in terms of LOOCV. The behavioral reference line shows how well one could do using information in the behavioral data. It was calculated using the mean position of the behavioral phase boundaries for each set of trials of a particular number of time points (varying from 9 to 26 time points). It represents the smallest possible prediction error we could achieve using the behavioral data alone. The HSMM‐MVPA analysis does not have this information but must discover what constitutes task boundaries and where they are.

The results show relatively good performance in a range from about .005 to .15 with best performance at roughly .05, which corresponds to the variance ratios between a white matter region and highly responsive brain regions. Performance at our chosen value of .1 is only marginally worse. All of these values yield performance well below the behavioral reference value.
